# Do physiological changes in fatty acid composition alter cellular ferroptosis susceptibility and influence cell function?

**DOI:** 10.1016/j.jlr.2025.100765

**Published:** 2025-02-26

**Authors:** Graeme I. Lancaster, Andrew J. Murphy

**Affiliations:** 1 Department of Immunometabolism, Baker Heart and Diabetes Institute, Melbourne, Victoria, Australia; 2 Department of Immunology, Monash University, Melbourne, Victoria, Australia; 3 Baker Department of Cardiometabolic Health, University of Melbourne, Melbourne, Victoria, Australia

**Keywords:** ferroptosis, polyunsaturated fatty acids, lipid peroxidation, cell death

## Abstract

Ferroptosis is an iron-dependent form of cell death driven by the excessive peroxidation of poly-unsaturated fatty acids (PUFAs) within membrane phospholipids. Ferroptosis is a hallmark of many diseases and preventing or inducing ferroptosis has considerable therapeutic potential. Like other forms of cell death, the pathological importance and therapeutic potential of ferroptosis is well appreciated. However, while cell death modalities such as apoptosis and necroptosis have critical physiological roles, such as in development and tissue homeostasis, whether ferroptosis has important physiological roles is largely unknown. In this regard, key questions for field are as follows: Is ferroptosis used for physiological processes? Are certain cell-types purposely adapted to be either resistant or sensitive to ferroptosis to be able to function optimally? Do physiological perturbations such as aging and diet impact ferroptosis susceptibility? Herein, we have reviewed emerging evidence that supports the idea that being able to selectively and controllably induce or resist ferroptosis is essential for development and cell function. While several factors regulate ferroptosis, it appears that the ability of cells and tissues to control their lipid composition, specifically the abundance of phospholipids containing PUFAs, is crucial for cells to be able to either resist or be sensitized to ferroptosis. Finally, aging and diets enriched in specific PUFAs lead to an increase in cellular PUFA levels which may sensitize cells to ferroptosis. Therefore, changes in dietary PUFAs or againg may impact the pathogenesis of diseases where ferroptosis is involved.

Ferroptosis is a form of cell death driven by excessive peroxidative damage to membrane phospholipids (PL), specifically those containing poly-unsaturated fatty acids (PUFAs) (1, 2, 3). Ferroptotic cell death occurs in many diseases and contributes to their pathology (4). Accordingly, preventing ferroptosis is a promising therapeutic strategy in such contexts. Alternately, triggering malignant cells to undergo ferroptosis is a promising approach in treating many cancer types and an area of intensive investigation (5). While other forms of regulated cell death are critical in maintaining cellular and organismal homeostasis (6), whether ferroptosis has major physiological roles is unclear. In this regard, recent evidence has highlighted important examples of why cells may need to either induce or resist ferroptosis to fulfill essential cellular functions.

While numerous factors influence a cell’s susceptibility to ferroptosis, including the levels of labile iron, redox status, and glutathione peroxidase (GPX)4 expression, the peroxidation and fragmentation of membrane PUFA-PLs is the defining step in the execution of ferroptosis (7, 8, 9). Accordingly, differences in membrane PUFA content, or potentially the ability to dynamically regulate cellular PUFA content, may be an important means by which cells can control their sensitivity to ferroptosis, for example, increasing PUFA-PL content to promote ferroptosis susceptibility or, vice versa, decreasing PUFA-PL content to reduce ferroptosis susceptibility. Indeed, it is well established that variance in PUFA content contributes to the differential susceptibility of different types of cancer cells to ferroptosis (10). However, whether differences in PUFA-PL content between healthy cell and tissue types influences their susceptibility to ferroptosis, and what the physiological role of such differences might be, is relatively unknown.

In this article, we firstly provide a brief outline of the essential regulators of ferroptosis, with a particular focus on the lipid metabolic enzymes that control the fatty acid (saturated/unsaturated/poly-unsaturated) composition of membrane PLs. For a more detailed description of the molecular underpinnings of ferroptosis, interested readers are referred to several excellent recent reviews (3, 4, 8). The main goal of this manuscript is to discuss the evidence that cells and tissues do indeed alter their PUFA-PL content to promote either susceptibility or resistance to ferroptosis, changes that are essential for cells and tissues to meet specific physiological requirements.

## Background on ferroptosis with a focus on PUFA-PLs

Dixon et al. were the first to formally identify ferroptosis as a unique form of cell death (1), with the ‘Fe’ in ferroptosis highlighting the essential role of iron (discussed below). In addition to iron, several other essential ingredients are required to initiate ferroptosis: (i) an excess production of reactive oxygen species, particularly the highly reactive hydroxyl radical (^•^HO), that overwhelms anti-oxidant defense mechanisms and (ii) enrichment of PUFA-PLs, particularly those PUFAs with ≥4 double bonds (e.g. 20:4, 22:6) (7). Such PUFAs are highly susceptible to H atom abstraction (the removal of a H atom from specific sites within the PUFA) by free radicals—the initiation step of lipid peroxidation. This causes the formation of a carbon-centered lipid radical (L^•^) which, upon reaction with O_2_, forms a lipid peroxyl radical (LOO^•^). LOO^•^ can abstract a H atom from the acyl chain of a neighboring PUFA within the cell membrane, creating a lipid-hydroperoxide (L-OOH) and another L^•^—the propagation step of lipid peroxidation (7). The excessive peroxidation of PUFA-PLs is the critical executioner step in ferroptotic cell death. While the exact basis by which PUFA peroxidation leads to ferroptotic cell death is unclear, there are likely several events that are important: (i) an increase in membrane tension leading to the activation of mechanosensitive channels, for example, Piezo-1, calcium influx, and the activation of the membrane pore–forming protein NINJ1 (8, 11, 12); (ii) oxidatively modified PUFAs, most notably PUFA-PL-OOH, readily decompose in the presence of iron thereby generating truncated PL species (typically 5 to 9 carbons in length). Such truncated PLs, which remain in cell membranes, are likely to significantly affect membrane properties and have been considered to be the major driver of membrane destabilization leading to ferroptotic cell death (2, 7); (iii) following PUFA-PL-OOH decomposition, both the truncated PL that remains in the membrane as well as the leaving group (e.g. 4-hydroxynonenal, malondialdehyde) are electrophilic and capable of forming adducts with nucleophilic amino acids in proteins (e.g. histidine residues) leading to protein dysfunction and potentially contributing to ferroptotic cell death (13).

Highly unsaturated PUFAs containing ≥4 double bonds are abundant in all cells where they are essential for many aspects of cell function. Indeed, a degree of lipid peroxidation occurs under “normal” conditions. Due to the propagative nature of lipid peroxidation reactions, a single L^•^ species can theoretically give rise to a wave of lipid peroxidation that spreads through a cell membrane. Accordingly, it is essential that cells can detoxify oxidatively modified PUFAs (e.g. those containing OOH, OO^•^, O^•^) to prevent the excessive lipid peroxidation that causes ferroptosis. Preventing excessive lipid peroxidation is achieved via several means, the most important of which is the selenocysteine-containing enzyme GPX4 (14, 15). GPX4 converts highly reactive lipid hydroperoxides (PUFA-PL-OOH) to the more stable lipid alcohols (PUFA-PL-OH) and thereby prevents the fragmentation of PUFA-PL-OOH (7). Studies using inhibitors of GPX4 (the classical pharmacological approach to trigger ferroptosis) or deletion of *Gpx4* underscore its importance in protecting cells against ferroptosis. As its name implies, GPX4 is dependent on glutathione, specifically its reduced form (GSH), for its activity (2). Accordingly, the ability to generate sufficient GSH to meet the needs of GPX4 is critical in preventing excessive lipid peroxidation. This is primarily achieved by the system X_c_^-^ antiporter, which imports cystine in exchange for glutamate, which is used to synthesize GSH (1, 8).

The second major means by which cells limit lipid peroxidation is via the use of radical trapping antioxidants (RTAs), which can be produced either endogenously or obtained from the diet (e.g. vitamins E and K) (7, 16, 17, 18). Regardless of their source, RTAs work by donating labile H atoms to PUFA-PL-OO^•^ (thereby generating a PUFA-PL-OOH which can be detoxified to a PUFA-PL-OH by GPX4) or PUFA-PL-O^•^ (to generate a PUFA-PL-OH) and thereby limit the propagation of lipid peroxidation reactions (7). The ferroptosis-suppressor protein (FSP)1 pathway is the major endogenous enzymatic pathway used by cells to generate RTAs (16, 19). FSP1 is an oxidoreductase that converts the oxidized forms of RTAs, in particular coenzyme Q10 but also vitamins E and K, to their reduced forms, that is, the forms of these molecules that contain labile H atoms that can be donated to detoxify lipid peroxides. The FSP1 pathway is particularly important in detoxifying lipid peroxides at the plasma membrane (19), a likely critical site in the ultimate demise of a cell via ferroptosis. As can be seen from the above, the inherent susceptibility of a cell to ferroptosis is influenced by several endogenous factors, for example, the availability of labile iron, the redox status of the cell, PUFA-PL abundance, but can also change contextually, for example, in response to activation, in certain disease states.

## Enzymatic regulation of PUFA-PL abundance

Given the central role of PUFA-PLs in ferroptosis, it is no surprise that lipid metabolic enzymes that influence the abundance of PUFA-PLs in cell membranes are key regulators of the cellular susceptibility to ferroptosis. While the importance of lipids in ferroptosis has always been appreciated, the observation that cells lacking ACSL4 are largely resistant to ferroptosis and the identification of specific pro-ferroptotic PUFA-PL substrates (notably phosphoethanolamine [PE] 18:0/20:4) confirmed the essentiality of PUFA-PLs in ferroptotic cell death (9, 20). ACSL4 converts free PUFAs with ≥4 double bonds to their CoA-ligated forms, which is essential for their incorporation into lyso-PLs to form PUFA-PLs. Accordingly, PLs from ACSL4-deficient cells have markedly reduced levels of PUFAs with ≥4 double bonds (e.g. 20:4, 22:6) (9, 20, 21). Relatedly, LPCAT1, MBOAT1/2, and LPCAT3 influence ferroptosis susceptibility through their ability to acylate (or re-acylate) lyso-PLs with saturated fatty acids, mono-unsaturated fatty acids (MUFAs), or PUFAs, respectively, and thereby control the acyl-chain composition of cellular membranes (22, 23, 24). Thus, for example, while MBOAT1/2 and LPCAT1 catalyze the formation of MUFA-PL and SFA-PL, respectively, and thereby promote ferroptosis resistance, inhibition of either MBOAT1/2 or LPCAT1 activity promotes ferroptosis susceptibility due to an increase in the proportion of PUFA-PL within cell membranes (23, 24). Accordingly, cell- and tissue-specific expression of these and other lipid metabolic enzymes establishes cellular and tissue lipid composition, with variance in the expression of these enzymes being essential to establish cellular- and tissue-specific lipidomes. Such differences are potentially a means by which specific cells and tissues may alter their susceptibility to ferroptosis.

## Physiological differences in PUFA-PL abundance influences ferroptosis susceptibility

**Fig. 1:**
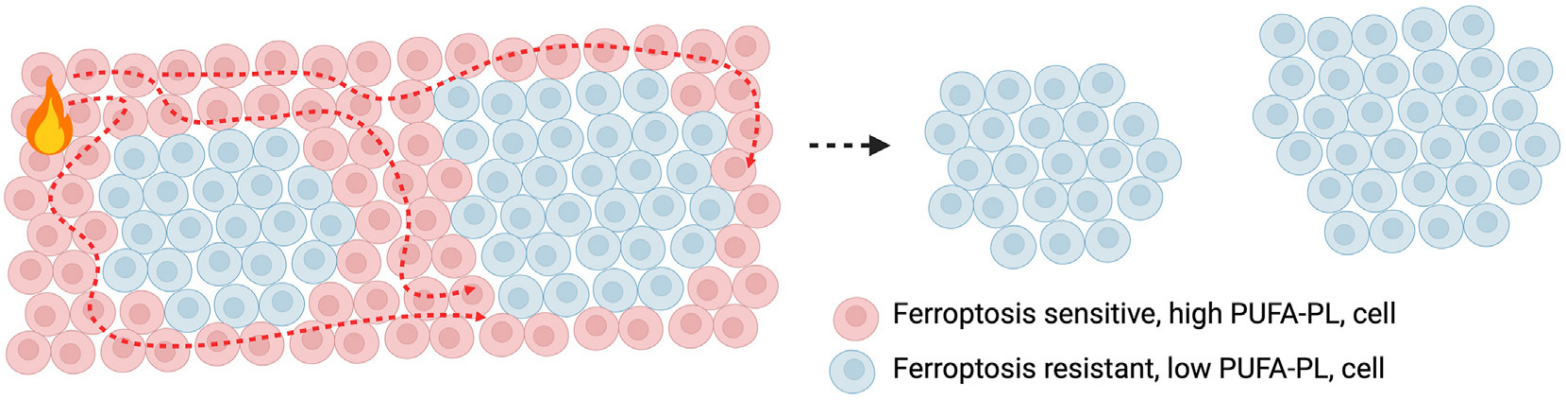
PUFA content allows for controlled propagation of ferroptosis. Ferroptosis has been shown to propagate through cell populations in a wave-like manner, whereby factors released from a ferroptotic cell triggers ferroptosis in neighboring cells. As a result, ferroptosis occurring in a specific cell can spread through an entire population of cells. Importantly, ferroptotic waves can be prevented by the creation of barriers. Such barriers can be created in vivo by altering the PL-PUFA content of cells, thereby creating areas that are sensitive to ferroptosis (high PL-PUFA content) and resistant to ferroptosis (low PL-PUFA content). Evidence suggests that such a process is used to remove unwanted cells during development and thereby aiding in tissue sculpting. The nature of the signals that trigger ferroptosis in such contexts are unclear.

By removing unwanted, damaged, or infected cells, cell death, particularly apoptosis, is essential for organismal development and homeostasis, for example, in tissue sculpting during embryogenesis and removing self-reactive T cells during T cell development (25). Several aspects of apoptosis make it ideally suited to such tasks: (i) it is nonlytic, that is, the cell membrane is not compromised and is therefore largely immunologically silent; (ii) apoptosis is genetically regulated and highly controlled, meaning that only cells that are targeted to undergo apoptosis die, there is little collateral damage to cells neighboring those undergoing apoptosis (25). Whether ferroptosis plays a significant role in organismal development and homeostasis has been an important question for the field, and some lines of evidence indeed suggest such a role. However, several features of ferroptotic cell death suggest it may not be suitable for the targeted removal of cells during developmental and homeostatic processes. Firstly, ferroptosis lacks the precise genetic and biochemical regulation of apoptosis. While cells possess multiple means to detoxify PUFA-PL-OOH and lipid radicals, and thereby prevent excess lipid peroxidation and ferroptosis, it is currently not known if specific signals (either internal or external – akin to the intrinsic and extrinsic pathways of apoptosis) can trigger ferroptosis in a deliberate and selective manner. Instead, the excessive lipid peroxidation that underlies ferroptosis is the result of a complex metabolic interplay between pro-ferroptotic (e.g. elevated labile iron, increased ROS, high cellular PUFA levels) and anti-ferroptotic (e.g. robust activity of lipid peroxide detoxification pathways, high MUFA levels, low labile iron, low ROS) factors, rather than a precise signal. Secondly, ferroptosis is a lytic form of cell death and the pores formed in the plasma membrane of ferroptotic cells allow for the relatively uncontrolled release of cellular contents (12). This means that ferroptosis is an inflammatory cell death, likely capable of affecting neighboring cells (26). Finally, there is evidence that ferroptosis can be “transmitted” between cells (27) (i.e. a ferroptotic cell can trigger ferroptosis in a neighboring cell), raising the issue of how such cell death can be effectively controlled. Accordingly, it seemed unlikely that ferroptosis would be a suitable cell death modality for the developmentally- and/or homeostatically-required removal of large numbers of unwanted cells. However, a recent study demonstrates that ferroptosis can spread through a cell population in a controlled manner through a process known as trigger waves (27). Specifically, Ho *et al.* (27) find that once initiated, ferroptosis can spread through a population of cells using trigger waves, whereby soluble factors released from a ferroptotic cell triggers ferroptosis in neighboring cells, which ultimately propagates through an entire cell population. Crucially, it is demonstrated that ferroptotic trigger waves occur during morphogenesis and contribute to the loss of cells required for sculpting the developing avian limb (Fig. 1). How can such a process be spatially controlled to ensure that only the intended cells are lost? Ho *et al.* firstly demonstrate that physical segregation stops ferroptotic trigger waves, but this is unlikely to be important within tissues in vivo. Remarkably, it is shown that variations in PUFA-PL abundance between regions of the developing tissue provide a means to control ferroptotic trigger waves, allowing their propagation in regions abundant in PUFA-PLs, but halting them in areas low in PUFA-PLs, and thereby allowing for the removal of large numbers of unwanted cells and sculpting the tissue to achieve its required morphology (27).

The work of Ho *et al.* is conceptually important as it demonstrates that varying the abundance of PUFA-PLs to influence ferroptosis susceptibility is a bona fide means to regulate critical physiological processes. An important question raised by this work is—is this mechanism more widely used or is it restricted to tissue morphogenesis during development? Our interest in this question stemmed from our work characterizing the lipidome of the human and mouse immune system (21). In this study, we assessed the levels of >500 individual lipid species across 16 human and 8 mouse immune cell types. Among the many differences in immune cell lipid composition we observed, one of the most striking was in the proportion of PUFA-PLs, particularly 20:4- and 22:6-containing PUFA-PLs. Specifically, lymphoid cells (various T cell and B cell subsets) had the highest proportions of PLs containing 20:4 (enriched within PC and PE PL classes) and 22:6 (enriched within PE(P) and PS PL classes), while myeloid cells, in particular neutrophils, had the lowest levels (21). These findings suggested that previously described differences in the susceptibility of lymphoid and myeloid cells to ferroptosis may be due to differences in the cellular proportion of PLs containing 20:4 and 22:6. Indeed, reducing the proportion of PLs containing 20:4 and 22:6 protected T cell from ferroptosis, while increasing the proportion of 20:4 and 22:6 PUFAs triggered ferroptosis in neutrophils (21).

While differences in PL acyl chain composition influences immune cell susceptibility to ferroptosis, whether immune cells deliberately alter their PL acyl chain composition to alter ferroptosis susceptibility, or whether differences in PL acyl chain composition are required for other aspects of immune cell function and changes in ferroptosis susceptibility are an unintended consequence, is unclear. However, several lines of evidence suggest that some immune cells may indeed deliberately alter their PL acyl chain composition to alter susceptibility to ferroptosis. For example, neutrophils are remarkably resistant to ferroptosis, and this appears in part to be due to their low level of PLs containing 20:4 and 22:6. But why might neutrophils need to be resistant to ferroptosis? Neutrophils are the immune systems key sentinels, patrolling the vasculature and responding to infection and tissue damage (28). These environments are highly oxidative and are enriched in iron, O_2_, ROS, and lipid radicals that can promote lipid peroxidation and ferroptosis. Accordingly, it would make intuitive sense that neutrophils must be inherently resistant to lipid peroxidation and ferroptosis to enable them to successfully function in such environments. Moreover, one of the most important aspects of neutrophil biology is their capacity to rapidly produce large quantities of the superoxide anion (O^-^_2_), the oxidative burst, via the phagocyte-specific form of NADPH oxidase (NOX) – NOX2 (29). O^-^_2_ is rapidly converted to H_2_O_2_, which, as discussed above, upon reaction with iron can form HO^•^, a critical initiator of lipid peroxidation. Again, it seems logical that neutrophils must be able to resist the initiation of lipid peroxidation from these endogenously produced ROS, otherwise their viability and function would be severely compromised. Therefore, given the likely importance of resisting lipid peroxidation for neutrophil functionality, balancing their acyl chain profile towards PLs that contain relatively low levels of 20:4 and 22:6 may indeed be a feature of the neutrophil lipidome that has evolved specifically to enable neutrophils to resist lipid peroxidation and ferroptosis. Consistent with this idea, the proportion of PLs containing 20:4 progressively and markedly declines during neutrophil development, while 22:6 PUFAs are already relatively low in hematopoietic stem and progenitor cells and decrease only slightly during neutrophil development (21). Experimentally, we found that supplementing neutrophils with high levels of 20:4 and 22:6 PUFAs, thereby increasing the abundance of PLs containing 20:4 and 22:6, exacerbates lipid peroxidation and leads to ferroptosis (independent of GPX4 inhibition) (21). Notably, neutrophils activated with PMA, a potent stimulator of ROS production via the oxidative burst, displayed a modest increase in lipid peroxidation, but did not succumb to ferroptosis. However, supplementing neutrophils with low levels of 20:4 and 22:6 PUFAs prior to activating them with PMA increased lipid peroxidation and resulted in marked ferroptosis (21). These findings further support the idea that the low levels of PLs containing 20:4 and 22:6 protects neutrophils from excessive lipid peroxidation and ferroptosis and this may be an important feature of neutrophils that allows them to function effectively.

While neutrophils are generally resistant to ferroptosis, under specific pathological settings, neutrophils are indeed susceptible to ferroptosis. It has been shown that neutrophils undergo ferroptosis in disorders such as systemic lupus erythematosus (30), infection (31), and the tumor microenvironment (32). Neutrophil ferroptosis in these settings contributes to disease pathogenesis. For example, in the context of cancer, the contents released from ferroptotic neutrophils within the tumor microenvironment are immunosuppressive to T cells, limiting their ability to kill their target cells (32). In systemic lupus erythematosus, ferroptosis appears to be responsible for neutropenia, largely triggered by autoantibodies and IFN-α (30). A function largely unique to neutrophils is their ability to release their DNA contents (neutrophil extracellular traps; NETs) in a process known as NETosis. This process requires rapid and vast dissociation of the cell membrane as the spatial requirements to deploy NETs is significant. During modeled infection, where the release of NETs helps to bind and neutralize bacteria, neutrophils sense pathogens via TLRs which promotes the generation of ROS and chromatin decondensation, while they also bind activated platelets which deposit iron to induce lipid peroxidation. Importantly, inhibiting lipid peroxidation resulted in reduced NETs (31). Thus, it appears that at times, NETosis is linked to lipid peroxidation, but it is important to note that this should not assume ferroptosis is also an occurrence. Adding complexity to the requirement of lipid peroxidation of PUFAs to NETosis is a study exploring the sensitivity of neutrophils from people with type 2 diabetes that have been supplemented with PUFAs (EPA & DHA; highly susceptible to lipid peroxidation) and vitamin D3 and which resulted in the prevention of PMA-induced NETs. Therefore, we suggest that detailed mechanistic studies exploring the alteration of the neutrophil cell membrane lipid composition and lipid peroxidation to facilitate NETosis are required.

In contrast to neutrophils, T cells are highly susceptible to ferroptosis (21). Notably, the vulnerability of T cells to ferroptosis has been linked with impaired antitumor T cell responses (32, 33, 34). T cells have relatively high levels of PLs containing 20:4 and 22:6 PUFAs and altering PL acyl chain composition towards a more MUFA-enriched profile protects T cells from ferroptosis (21). However, whether T cells bias their PL acyl chain profile in favor of PLs containing 20:4 and 22:6 PUFAs to purposely alter their ferroptotic potential, or whether their susceptibility to ferroptosis is an epiphenomenon arising from other requirements for 20:4 and 22:6 PUFAs in T cell function, is unknown. Why might T cells need to be susceptible to ferroptosis? The negative selection (elimination) of self-reactive T cells by apoptosis during T cell development is essential to maintain immunological tolerance. The levels of PLs containing 22:6 increase during T cell development (21), so might ferroptosis also contribute to the thymic development and maturation of T cells? T cells lacking *Gpx4* appear to develop and mature normally within the thymus, suggesting that ferroptosis does not contribute to thymic T cell development (35). The susceptibility of T cells to ferroptosis may play a small role in the aging-associated loss of CD4 CD8 double-positive thymocytes (36). Interestingly, the loss of *Gpx4* does lead to reduced levels of CD8 T cells at peripheral sites (lymph nodes, spleen) and markedly lower CD4 and CD8 T cells following viral and parasitic infection, indicating that resistance to ferroptosis is essential for optimal T cell responses (35). The above studies collectively demonstrate that T cells must be able to resist ferroptosis to function effectively and that their high endogenous levels of 20:4- and 22:6-containing PUFA-PLs are likely essential for T cell function rather being required to promote a state of ferroptosis susceptibility. Indeed, T cells lacking *Acsl4* are resistant to ferroptosis but have impaired antitumor CD8 T cell responses (37), demonstrating the essentiality of 20:4- and 22:6-containing PUFA-PLs in T cell function.

Follicular helper T (T_FH_) cells are a subset of T cells that activate B cells within germinal centers to promote the formation of long-lived memory B cells and high affinity plasma cells. Recently, it was shown that the loss of *Gpx4* leads to markedly reduced numbers of T_FH_ cells and impaired humoral immune responses following immunization (38), demonstrating once again that resistance to ferroptosis is essential for T cell function. Intriguingly, it was also shown that T_FH_ cells from WT mice show hall marks of ferroptosis, that is, increased lipid peroxidation, following immunization (38). TCR engagement appears to be critical in increasing cellular ROS and thereby increasing lipid peroxidation and ferroptosis in T_FH_ cells (38). T_FH_ cells partake in multiple rounds of engagement with germinal center B cells and, therefore, are likely subject to repeated increases in ROS. We postulate that after a certain number of T_FH_ cell-germinal center B cell interactions have occurred, a threshold of lipid peroxidation is reached by the T_FH_ cells, triggering their ferroptosis, and thereby serving as a natural means of limiting T_FH_ cell responses. While neither we nor others have specifically examined the PUFA-PL profile of T_FH_ cells, our previous data indicates that high levels of PLs containing 20:4 and 22:6 PUFAs are a general feature of T cells (21), and therefore this PUFA-PL phenotype may underpin the initiation of ferroptosis in T_FH_ cells.

## Aging and ferroptosis

The oxidative stress theory of aging is the most widely accepted explanation of the variation in maximum life span potential between different species (6). In this model, longevity is thought to be closely linked to the production of oxygen free radicals (originating primarily from mitochondrial activity) and consequent damage to various biomolecules (e.g. DNA, proteins, lipids) (6). An important refinement to this model was the discovery that membrane PL fatty acid composition varies markedly between species and that there is an inverse relationship between maximum life span and the PUFA content of membranes, that is, higher PUFA content is linked to a lower maximum life span due to the inherent higher peroxidation potential of PUFAs, particularly PUFAs with ≥4 carbon-carbon double bonds, and the consequences thereof (6). Whether an increase in ferroptosis per se, also contributes to the interspecies variation in maximal life span potential is unclear and potentially difficult to untangle due to the close relationship between lipid peroxidation and ferroptosis and that there are currently no markers that uniquely distinguish between lipid peroxidation and ferroptosis. Interestingly, we have shown that murine T cells, which have an approximately 2-fold higher proportion of 22:6-containing PLs than human T cells, are markedly more sensitive to ferroptosis than human T cells (21). While interspecies differences in PUFA-PL content contribute to maximal lifespan potential, it was recently demonstrated that PUFA content also changes during the normal aging process (39). Specifically, it was demonstrated that PUFA content and the PUFA/MUFA ratio increases across multiple tissues as mice age. Interestingly, in a separate study, using an antibody to identify ferroptotic cells, it was shown that the prevalence of ferroptosis across multiple tissues increased during aging in rats (40). While this antibody was raised to recognize 4-hydroxy-2-nonenal–modified proteins and therefore does not absolutely discriminate between lipid peroxidation (which can occur without inducing cell death) and ferroptosis. Taken together, these studies suggest that aging-induced changes in tissue/cell PUFA content sensitizes to lipid peroxidation and potentially ferroptosis, particularly if there were further stress on the cell that would occur with diseases of the aged.

## Diet and ferroptosis

There has long been an interest in modifying diets or administering supplements to influence dietary PUFA intake. This is particularly evident in the context of cardiovascular disease, where fish oil supplements, high in n-3 PUFAs (DHA and EPA), have long been used for their beneficial effects in reducing plasma cholesterol but afforded little benefit in respects to lowering CV events (41, 42, 43, 44). Importantly, dietary PUFAs are rapidly incorporated into human and murine cells (45, 46). Therefore, the obvious link would be to suggest that given the abundance of PUFA-containing PLs has a major influence on ferroptosis, diets enriched in PUFAs may impact ferroptosis susceptibility. When fed a diet enriched in fish oil, mice lacking one copy of *Gpx4* specifically within intestinal epithelial cells displayed aspects of Crohn’s disease, including small intestinal inflammation and neutrophil recruitment, and had signs of lipid peroxidation within epithelia (47). Notably, this phenotype is not observed in WT mice, suggesting that a fish oil–enriched diet alone is very unlikely to promote ferroptosis, and that some second signal, in this case a loss of one copy of *Gpx4* (i.e. weakened anti-oxidant response), is required to initiate ferroptosis under such conditions. Studies such as this would therefore caution the use of PUFA-enriched/supplemented diets in chronic diseases where anti-oxidant defenses might be compromised, particularly in the aged as noted above.

However, there is also potential to harness dietary PUFAs to limit disease. Indeed, a fish oil–enriched diet was shown to prevent colorectal tumor growth and enhanced survival (48). Importantly, the fish oil–enriched diet increased markers of lipid peroxidation, indicative of ferroptosis, within the tumor tissue, and these positive effects could be reversed when an inhibitor of ferroptosis (Ferrostatin-1) was administered, suggesting the reduction in tumor growth by PUFA-enriched diets was through ferroptosis (48). A similar finding was also observed in pancreatic cancer models (49). Alternatively, approaches to force cancer cells mobilize stored PUFAs for incorporation into membranes through limiting extracellular availability of lipid has also been shown to promote ferroptosis (50). Therefore, it appears that several approaches could be employed to increase membrane PUFA-PL content in cancer cells to sensitize them to ferroptosis. Whether these mechanisms activated in cancer cells are also activated in normal physiology will be interesting to discover. Nonetheless, increasing the PUFA content of cancer cells through dietary manipulation is an exciting approach to treat specific cancers; however, there may be some caveats to this approach which require careful consideration.

T cell–dependent immunity is essential for the eradication of many cancers, yet several papers have recently shown that T cells undergo ferroptosis within the tumor microenvironment, potentially due to increased T cell PUFA uptake, thereby limiting antitumor T cell responses (33, 34). Accordingly, a diet enriched in PUFAs could also increase T cell PUFA content and predispose these cells to be further sensitized to ferroptosis within the tumor microenvironment, thereby further limiting antitumor T cell immunity. Additionally, in PUFA-enriched tumors, IFNγ release by T cells synergizes to induce tumor cell ferroptosis (51). Whether the lipid peroxides released by the dead tumor cells could also propagate ferroptosis in the engaged T cell is yet to be explored.

**Fig. 2:**
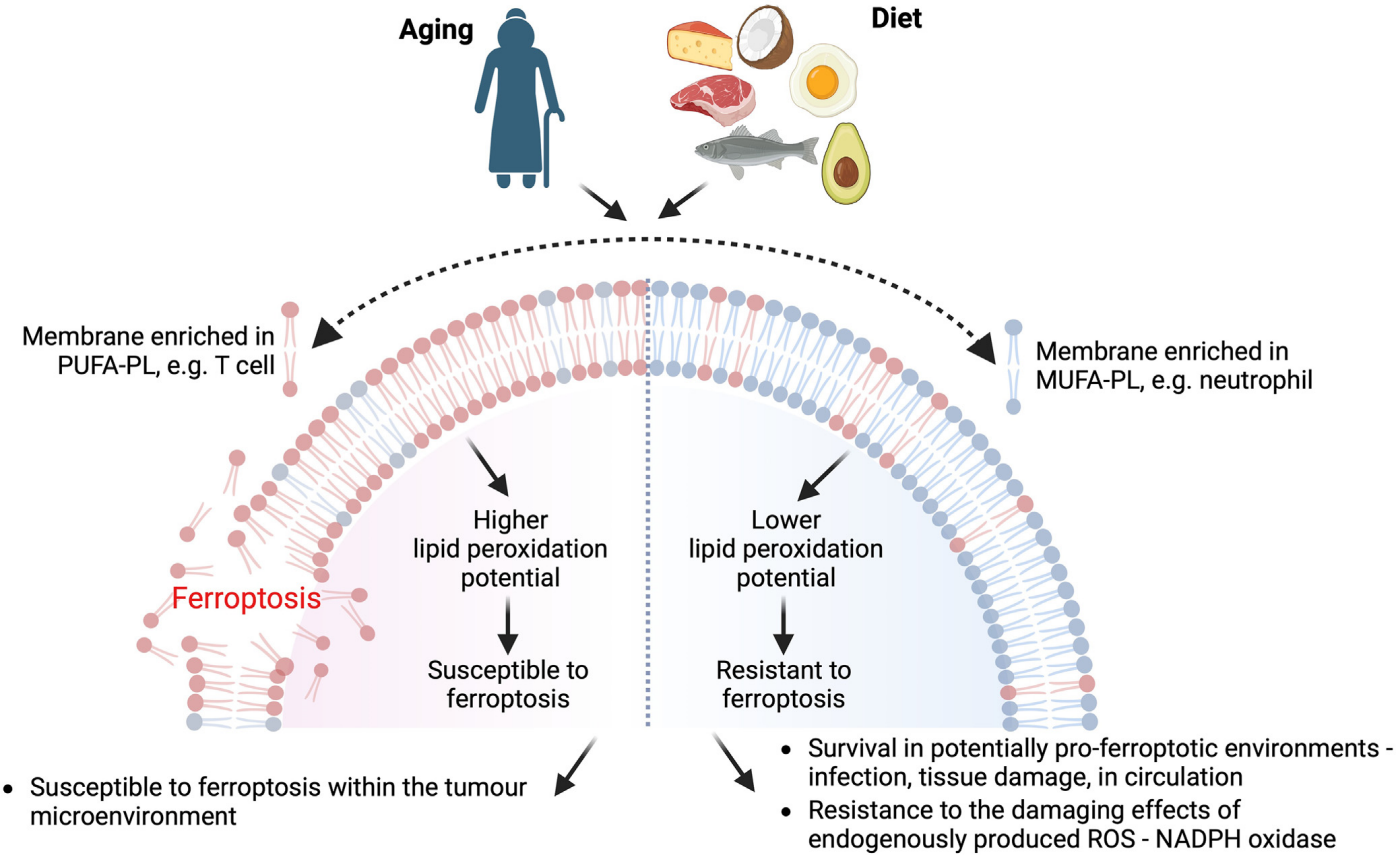
PUFA-FL content of cell regulates susceptibility to ferroptosis. Cell membranes enriched in PUFA-PL increased the susceptibility of the cell to ferroptosis, whereas cell membranes that contain low levels of PUFA-PL, generally offset with higher levels of MUFA-PL have a reduced susceptibility to ferroptosis. Membrane PUFA- and MUFA-PL content can be dynamically altered by factors such as aging and diet, impacting the ferroptotic susceptibility of the cell and potentially contribute to disease or aid therapies.

Furthermore, ferroptotic cell death occurs in many diseases and contributes to disease pathology; it is therefore possible that in specific disease contexts, the consumption a diet enriched in PUFAs, either through consumption of oily fish or via supplements containing fish oil, could exacerbate disease pathology by increasing ferroptosis susceptibility. Finally, given the strong antiferroptotic effects of MUFAs in vitro (52), it may be possible that consuming diets low in PUFAs and enriched in MUFAs could limit ferroptosis susceptibility in vivo and, once again, this could be exploited for therapeutic gain in certain disease contexts (a summary of the above discussion is shown in Fig. 2).

## Conclusion

Since its discovery in 2012, enormous progress has been made in understanding the fundamental biology of ferroptosis as well as how to use this information therapeutically, particularly in cancer. Whether ferroptosis is used to control physiological processes has been unclear, although certain examples have suggested such a role. It is now evident that triggering ferroptosis is used to control specific developmental processes and that cells that have to function in proferroptotic environments (e.g. sites of infection or tissue damage) are adapted to resist ferroptosis. Importantly, both resistance and sensitization to ferroptosis are achieved by altering the cellular content of PUFA-PLs. While the idea that PUFA-PLs can be used to alter ferroptosis susceptibility in a context- and cell-dependent manner is well accepted, in vivo implementation will require approaches that can alter PL-PUFAs in a cell/tissue selective manner. The abundance of PUFA-PLs can be controlled genetically by altering the expression of specific enzymes such as *ACSL4*. Indeed, melanoma patients with high tumor *ACSL4* levels had improved survival and response to immunotherapy (51). We eagerly await future research that further explores potential physiological roles for ferroptosis and hypothesize that variance in PUFA-PL content is likely to be an important player in such processes. We suggest that mechanistic insight to these roles will yield novel ways to manipulate cells/tissue for therapeutic gain centered around ferroptosis. Finally, it will also be exciting to see how simple physiological interventions such as alterations to diet can be used to shape cellular lipid content and ferroptosis susceptibility in certain disease contexts.

## Conflicts of interest

The authors declare that they have no conflicts of interests with the contents of this article.
